# Telomere Length of Circulating Leukocyte Subpopulations and Buccal Cells in Patients with Ischemic Heart Failure and Their Offspring

**DOI:** 10.1371/journal.pone.0023118

**Published:** 2011-08-18

**Authors:** Liza S. M. Wong, Jardi Huzen, Rudolf A. de Boer, Wiek H. van Gilst, Dirk J. van Veldhuisen, Pim van der Harst

**Affiliations:** 1 Department of Cardiology, University Medical Center Groningen, University of Groningen, Groningen, The Netherlands; 2 Department of Cardiology, VU University Medical Center, Amsterdam, The Netherlands; 3 Department of Psychiatry, University Medical Center Groningen, University of Groningen, Groningen, The Netherlands; Roswell Park Cancer Institute, United States of America

## Abstract

**Background:**

We aimed to find support for the hypothesis that telomere length (TL) is causally involved in the pathogenesis of ischemic heart failure (IHF). We measured TL in IHF patients and their high-risk offspring and determined whether mean leukocyte TL reflects TL in CD34+ progenitor. We additionally measured TL of offspring of patients and controls to examine heritability throughout different cell types.

**Methods and Results:**

TL was measured by qPCR in overall leukocytes, CD34+ progenitor cells, mononuclear cells (MNCs), and buccal cells in 27 IHF patients, 24 healthy controls and 60 offspring. TL in IHF patients was shorter than healthy controls in leukocytes (p = 0.002), but not in CD34+ cells (p = 0.39), MNCs (p = 0.31) or buccal cells (p = 0.19). Offspring of IHF patients had shorter TL in leukocytes than offspring of healthy subjects (p = 0.04) but not in other cell types. Controls and offspring showed a good within person correlation between leukocytes and CD34+ cells (*r* 0.562; p = 0.004 and *r* 0.602; p = 0.001, respectively). In IHF patients and offspring the correlation among cell types was blunted. Finally, we found strong correlations between parent and offspring TL in all four cell types.

**Conclusions:**

Reduced leukocyte TL in offspring of IHF subjects suggests a potential causal link of TL in ischemic heart disease. However, this causality is unlikely to originate from exhaustion of TL in CD34+ progenitor or MNC cells as their lengths are not well captured by overall leukocyte TL. Additionally, we found strong correlations between parent and offspring TL in all examined cell types, suggesting high heritability of TL among cell types.

## Introduction

Telomeres are the terminal ends of the DNA strands, and shorten during life because of incomplete DNA replication after cell cycling or damaging environmental factors. Cells with critically short telomeres become dysfunctional, and can eventually even go into apoptosis [1], [2]. Recently, telomere biology has been implicated in aging associated cardiovascular diseases. Most data has been generated on establishing the association between short mean overall leukocyte telomere length (TL) and ischemic heart disease (IHD) [3], [4], [5], [6]. In addition, it has been suggested that presumably healthy offspring of patients with ischemic heart disease already have shorter TL compared to healthy offspring of controls [7]. An open question remains whether telomere length is causally involved in the development of heart disease, and if so, what the underlying mechanism is. Short overall mean leukocyte telomere length has been viewed as a reflection of short telomere length in other cells, possibly of vascular progenitor cells, and thereby providing a link to an impaired vascular repair mechanism potentially causing ischemic heart disease.

To further dissect the association of ischemic heart disease with mean overall leukocyte TL we need to establish whether mean overall leukocyte TL is a reflection of TL in different cell types or whether it is more or less specific for leukocytes. Of particular interest in this regard are the CD34 positive (CD34+) cells as it is thought that these cells might be cardiovascular progenitor cells and play a role in cardiovascular repair [8], [9], [10]. Short TL in CD34+ cells might provide a mechanism for the association with IHD as their cellular dysfunction might impair cardiovascular repair. Furthermore, mean leukocyte telomere length has not been compared to non-circulating non-vascular cells and it is unknown whether leukocytes might merely be a reflection of overall TL of the whole body [11].

We have investigated telomere length in circulating leukocytes, CD34+ cells, mononuclear cells, and the non-systemic non-circulating buccal cells in patients with ischemic heart failure (IHF) – which is the most extreme phenotype of IHD – and compared them to healthy, age-matched controls. Since occurrence of IHD is highly familial and telomere length is an inheritable trait [12], [13], [14], we also aimed to determine whether telomere length in the different cell types is shorter in offspring of IHF patients compared to offspring of healthy controls.

## Methods

### Ethics statement

The study was approved by the local Medical Ethical Committee for human research of the University Medical Center Groningen (UMCG) and adheres to the Declaration of Helsinki. All participants were aged 18 or older and provided written informed consent for participation in this study.

### Study design

The Telosophy study is an observational, prospective case-control study. Groups consisted of 1) patients with stable ischemic heart failure (IHF) and 2) age and gender matched healthy controls. In addition, of both groups we included offspring; (3) offspring of IHF patients, and (4) offspring of healthy controls.

The inclusion criteria for the IHF patients were: presence of coronary artery disease (previous myocardial infarction and/or coronary revascularisation), left ventricular ejection fraction (LVEF) ≤40%, having heart failure for at least 6 months and being stable on optimal medication for heart failure for at least 4 weeks prior to the study visit, and having at least one healthy biological child that was willing to participate. Exclusion criteria were having had an ischemic cardiac event in the past 12 months and having severe cardiac valvular disease. Inclusion criteria for controls were being age- and gender- matched to the IHF patients, and having at least one healthy biological child who was willing to participate. Exclusion criteria for healthy controls were having known atherosclerotic disease or heart failure, a family history of premature cardiovascular disease, and having a partner (who is the biological parent of the participating offspring) with known cardiovascular disease. In addition, main exclusion criteria for all participants were having systemic inflammatory diseases, haematopoietic diseases, severe renal of liver disease, uncontrolled hypothyroidism, premature aging syndromes, or malignancies, which could all influence telomere length.

### Study visit

IHF patients were recruited from the outpatient clinic of the Universitair Medical Center Groningen in Groningen, the Netherlands. Healthy controls were recruited with local advertisements or screened at the pre-operative outpatient clinic for planned selective non-cardiovascular surgery (small plastic surgery, ophtalmic surgery or minor orthopaedic procedures).

Subjects underwent 1 study visit to assess medical and family history and life style factors and to undergo a physical exam. Venous blood samples (60 mL in collected in tubes precoated with EDTA and 2.5 mL in the PAXgene Blood RNA tube, catalogno. 762174, Qiagen, Venlo, The Netherlands) and a buccal mucosa cells (four buccal swabs (isohelix, SK-4)) samples were taken.

### Isolation of CD34+ cells

Peripheral blood mononuclear cells were obtained from venous whole blood after density gradient centrifugation (Ficoll Paque Plus, catalogno. 17-1440-02, GE Healthcare Europe GmbH, Diegem, Belgium). The mononuclear cell (MNC) fraction was incubated with magnetic beads conjugated to anti-CD34-antibody (CD34 Microbead Kit, catalogno. 130-046-702, Miltenyi Biotec, Bergisch Gladbach, Germany). Subsequently, CD34+ cells were obtained from the MNC fraction by magnetic bead cell selection (MidiMACS separator, Miltenyi Biotec, Bergisch Gladbach, Germany). Flow cytometric analysis of mononuclear cells showed that incubation with anti-CD34-antibody and magnetic beads increased the CD34+ cell isolation from 0.39% to 58.81% (data not shown).

### Telomere length measurement

DNA isolation of CD34+ cells, MNCs, and buccal swabs was performed according to manufacturer's protocol (NucleoSpin Tissue kit, catalogno. 740952, Macherey-Nagel/Bioké, Leiden, The Netherlands). Relative telomere length (TL) was assessed by monochrome multiplex quantitative PCR method, previously described in detail [15], [16], and expressed as the ratio of telomere (T) to reference copies (S), further called T/S ratio. Samples of IHF patients, controls, and both offspring groups were randomly assigned to the plates and plate positions, with each plate containing samples of all four groups. All samples were run in triplicate. Samples with a coefficient of variation larger than 0.10 were run again. No samples had a coefficient of variation larger than 0.10 after the second run.

Data were characterised as outliers when >4 times standard deviation and were excluded from all statistical analyses (N = 1).

### Statistical analysis

Skewed variables were natural log transformed to acquire normal distribution. Differences in means between the groups were tested with Student's T-test or Chi square test. Pearson (or Spearman) and linear regression techniques were used to asses associations between variables of interest. Multivariate linear regression analysis was used to make adjustments. All statistical analyses were performed in SPSS version 16.0 (SPSS inc. Chicago, Illinois). A two-sided p-value of ≤0.05 was interpreted to indicate statistical significance.

## Results

### Baseline characteristics

Baseline characteristics per group are presented in Table 1. All IHF patients had experienced previous myocardial infarction. On average, IHF patients were aged 69 years, had a mean LVEF of 27%, 85% was male, and were well treated.

**Table 1:** Baseline characteristics.

Patient characteristics	I: CHF patientsN = 27	II: Healthy controlsN = 24	P-value	III: Offspring of CHF patientsN = 29	IV: Offspring of healthy controlsN = 25	P-value
**Age (years)**	69±6.9	66±6.6	0.14	40±6.8	38±6.0	0.28
**Male (n (%))**	23 (85)	20 (83)	0.86	17 (59)	9 (36)	0.10
**Age of onset CHF (years)**	60±8.5	-	-	-	-	-
**Body mass index (kg/m^2^)**	28.0±5.0	25.3±3.0	0.04	27.2±4.5	24.0±2.4	0.002
**Left ventricular ejection fraction (%)**	27±8.2	-	-	-	-	
**NYHA class (n (%))**			-			-
**II**	12	-		-	-	
**III**	15	-		-	-	
**IV**	0	-		-	-	
**CRP (mg/L)**	0.0 [0.0–0.0]	0.0 [0.0–0.0]	0.38	0.0±0.0	0.0±0.0	0.55
**NTproBNP (pg/mL)**	665 [364–1524]	52 [35–63]	<0.001	24 [11–41]	19 [8–45]	0.97
**Heart rate (beats/min)**	66±8	67±9	0.76	69±13	71±10	0.47
**Blood pressure (mmHg)**						
**Systolic**	114±15	133±18	<0.001	123±18	121±17	0.64
**Diastolic**	68±10	80±11	<0.001	76±11	77±13	0.71
**Medical history (n (%))**						
**Myocardial infarction**	27 (100)	0 (0)	<0.001	0 (0)	0 (0)	1.00
**Hypertension**	4 (15)	5 (21)	0.57	2 (7)	1 (4)	0.64
**Diabetes mellitus**	8 (30)	1 (4)	0.02	1 (3)	0 (0)	0.35
**Atrial fibrillation/flutter**	11 (37)	0 (0)	<0.001	1 (3)	0 (0)	0.35
**Stroke**	3 (11)	0 (0)	0.09	0 (0)	0 (0)	1.00
**Hypercholesterolaemia**	13 (48)	0 (0)	<0.001	4 (14)	0 (0)	0.05
**Medication**						
**Beta-blocker**	26 (96)	3 (13)	<0.001	1 (3)	0 (0)	0.35
**ACE inhibitor and/or AII antagonist**	27 (100)	3 (13)	<0.001	1 (3)	1 (4)	0.92
**Aldosterone antagonist**	7 (26)	0 (0)	0.007	0 (0)	0 (0)	1.00
**Diuretic**	22 (81)	1 (4)	<0.001	2 (7)	1 (4)	0.64
**Statin**	24 (89)	1 (4)	<0.001	3 (10)	0 (0)	0.10
**Anticoagulant**	26 (96)	0 (0)	<0.001	0 (0)	0 (0)	1.00

Data is presented as mean ± standard deviation or number (%). CHF – chronic heart failure; MNC – mononuclear cells; CD34+ – CD34-positive cells; The body-mass index is the weight in kilograms divided by the square of the height in meters; NYHA – New York Heart Association functional class; CRP – C-reactive protein; NTproBNP – N-terminal proB-type Natriuretic Peptide; ACE – Angiotensin Converting Enzyme; AII – Angiotensin II.

Offspring of IHF patients had a higher BMI and tended to have more frequently hypercholesterolemia compared to offspring of controls.

### Telomere length in leukocyte subpopulations in patients, controls, and their offspring

**Figure 1:**
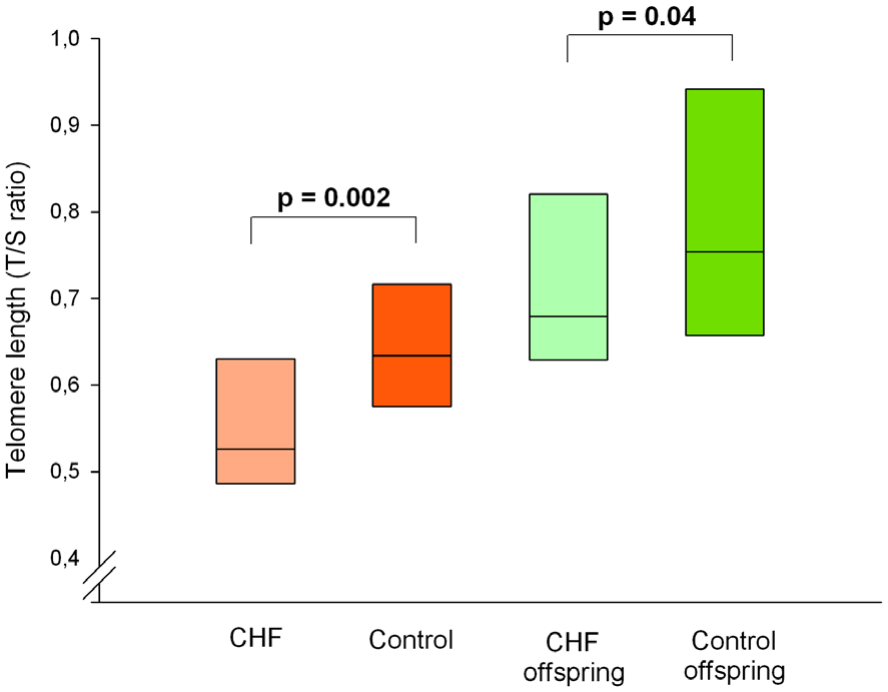
Mean leukocyte telomere length of all groups.

In concordance with previous findings [17], [18], we observed shorter leukocyte TL in IHF patients compared to controls (Figure 1). We also observed shorter leukocyte TL in offspring of IHF patients compared to offspring of healthy controls (Figure 1). One of the aims of this study was determining whether telomere length of CD34+ cells is different in IHF patients compared to healthy controls. We did not find a difference in TL between IHF patients and controls in CD34+ cells (mean TL ± standard deviation 0.56±0.17 and 0.60±0.14, respectively, p = 0.39). Also the TL of MNCs was similar (0.46±0.12 and 0.49±0.10, respectively, p = 0.31) as were TL of buccal cells (0.97±0.27 and 1.04±0.19, respectively, p = 0.19). In offspring, TL observed in CD34+ cells (0.66±0.16 and 0.71±0.16; p = 0.22), MNCs (0.62±0.17 and 0.62±0.17; p = 0.96), and buccal cells (1.17±0.42 and 1.21±0.25; p = 0.47) were comparable.

These results clearly indicate that there is no significant difference in CD34+ cell TL between IHF patients and controls. CD34+ cell dysfunction as a result of short TL is therefore an improbable player in the pathogenesis of IHF.

### Strong correlation between leukocyte and CD34+ in controls and their offspring, but not IHF patients and their offspring

Another aim of this study was to investigate whether within one individual the more frequently reported leukocyte TL mirrors TL of the cells of our interest, the CD34+ cells. Therefore, we examined the correlation between TL of leukocytes and CD34+ cells. Interestingly, we found strong correlations between TL of leukocytes and CD34+ cells in controls and their offspring but not in IHF patients or their offspring (Table 2)

**Table 2:** Correlation coefficients between Cd34+ cells and other cell types within patients, controls, and offspring.

	Correlation coefficient *r*	P value
**CHF patients**	0.313	0.137
**Healthy controls**	0.562	0.004
**Offspring of CHF patients**	0.210	0.324
**Offspring of healthy controls**	0.602	0.001

### Strong correlations between parent and offspring TL in all cell types

**Figure 2:**
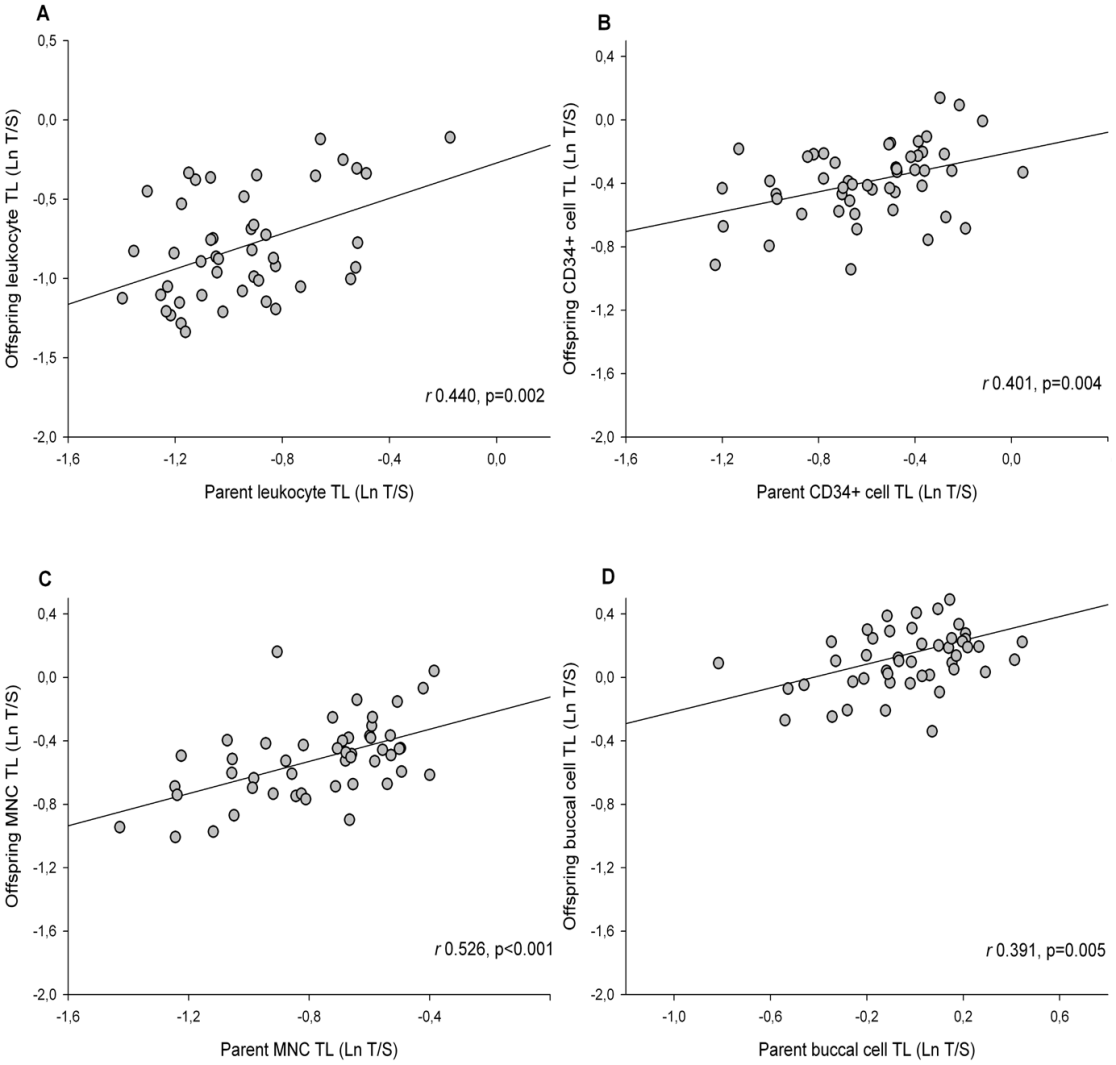
Correlations between parent and offspring telomere length in leukocytes (A), CD34+ cells (B), mononuclear cells (C), and buccal cells (D).

We found a positive association of TL between parent and offspring in leukocytes (correlation coefficient *r* 0.440, p = 0.002). We also observed this association in CD34+ cells (*r* 0.401, p = 0.004), MNCs (*r* 0.526, p<0.001), ), and in buccal cells (*r* 0.391, p = 0.005) (Figure 2). These associations remained significant after correction for age of parent and offspring (for leukocytes *r* 0.448, p = 0.004), for MNCs *r* 0.547, p<0.001; for CD34+ cells *r* 0.397, p = 0.004; for buccal cells *r* 0.368, p = 0.010). We could not validate earlier findings suggesting an association of paternal age at time of birth with offspring telomere length [13].

## Discussion

Shorter mean leukocyte TL is a remarkable and consistent finding in subjects with ischemic heart disease, but the reason is not known. Nevertheless, short overall mean leukocyte telomere length has been viewed as a reflection of short telomere length in other cells, possibly of vascular progenitor cells, and thereby providing a link to an impaired vascular repair mechanism potentially causing ischemic heart disease. We indeed observed a good correlation between overall mean leukocyte telomere length and CD34+, MNCs and buccal cells in healthy subjects and also in their offspring. However, these high intra-individual correlations were lost in subjects with IHF and their offspring. The major difference in telomere length between IHF patients and controls was observed in the overall leukocyte pool, not specifically in CD34+, MNCs or buccal cells as a source of non-blood derived cells. We confirmed earlier findings, suggesting shorter leukocyte telomere length in offspring of patients with coronary artery disease versus offspring of healthy controls [7]. Finally, we confirmed the strong associations between parent and offspring TL in all four cell types we examined.

The comparable TL of CD34+ cells in cases and controls and both offspring groups are consistent with earlier findings [19] and strongly suggest that telomere shortening of CD34+ cells is not a major player in the pathophysiology of IHD. If there is any role of TL in this process – the consistent finding of shorter leukocyte TL in IHD patients does support this allegation – it would rather involve other leukocyte populations. It is known that leukocyte function changes with aging. In the elderly, specific immune responses might be diminished, but many other functions are unchanged or even augmented compared to young persons [20]. A state of low-grade chronic inflammation has been recognized in elderly, which makes them prone to chronic inflammatory diseases, including atherosclerosis [20]. Macrophages, and to a lesser extent other leukocytes, catalyse the process of atherosclerosis by eliciting an enhanced systemic inflammatory response, possibly through increased oxidative stress [20], [21], [22]. Interestingly, it has been demonstrated that macrophages with short telomeres are more susceptible to damage from oxidative stress, and in addition have higher intracellular concentrations of oxidative stress molecules [23], which are suggested to be a driving force in the development and progression of atherosclerosis [21]. These data strongly suggest that aging – chronologically or biologically – of leukocytes can possibly augment their unbeneficial contribution to IHD.

A secondary focus of our study was the heredity of TL traits among cell populations. Heritability of mean leukocyte telomere length has been demonstrated previously [12], [14], [24]. We now add to this the heritability patterns in TL derived from different cell fractions. A tissue specific TL regulation has been suggested, since TLs differ between different types of tissue [11]. In addition, external influences on TL are acknowledged. For instance, it has been shown that vascular endothelial cells that endure more hemodynamic sheer stress have shorter telomeres than endothelial cells in low pressure arteries [25]. Also, oxidative stress is a well-known factor that causes telomere shortening [26]. Our results indicate that despite external influences and tissue specific TL regulation, TL is a highly inheritable trait throughout different cell types.

### Strengths and limitations

Our study is strengthened by the fact that we not only examined TL of IHF patients and controls, but also of their offspring. Our finding that TL in healthy offspring of IHF patients is shorter than in healthy offspring of controls supports the hypothesis that, next to the genetic predisposition to coronary artery disease deriving from specific genes, TL might also be a factor contributing to familial predisposition to IHD. Furthermore, we separated leukocyte cell fractions to determine differential expression of TL. This is significant, since knowledge on which specific cell types contribute to the shorter mean leukocyte TL is necessary in order to identify a potential mechanism underlying the association between TL and IHD. To our knowledge, our study is the first study that investigated both heritability features and differential expression of TL in leukocyte subpopulations. Also, our study was a prospective study, meaning all measures were taken in advance to preserve blood and tissue samples taken from participants, which contributed to high accuracy of experimental procedures and laboratory measurements.

We also have to acknowledge some limitations. The separation of white blood cells was limited to MNCs and CD34+. Because of potential functional relevance, our main interest was the CD34+ cell population. However, we demonstrated that the CD34+ cell population is not the cell population that drives the difference in leukocyte TL between IHF patients and controls. This difference must thus lie in other leukocyte cell populations that we did not separately analyse in this study.

### Conclusions

In conclusion, we found that TL is shorter in leukocytes of IHF patients and their healthy offspring compared to healthy controls and their healthy offspring. This is supporting evidence for a causal role of TL in familial predisposition to ischemic heart disease. The fact that we did not find a difference in TL of CD34+ cells between IHF patients and healthy controls, suggests that these cells are not involved in the potential mechanism linking short TL and ischemic heart disease. Furthermore, we found good correlations between parent and offspring TL in all cell types we examined, indicating a strong inheritance pattern of TL. Evidence for a causal role of TL in ischemic heart disease will be helpful in finding new therapeutic targets.
